# Contribution of Heterotrophic Diazotrophs to N_2_ Fixation in a Eutrophic River: Free-Living vs. Aggregate-Associated

**DOI:** 10.3389/fmicb.2022.779820

**Published:** 2022-02-14

**Authors:** Eyal Geisler, Eyal Rahav, Edo Bar-Zeev

**Affiliations:** ^1^Zuckerberg Institute for Water Research, The Jacob Blaustein Institutes for Desert Research, Ben-Gurion University of the Negev, Sde Boker, Israel; ^2^Israel Oceanographic and Limnological Research, National Institute of Oceanography, Haifa, Israel

**Keywords:** diazotrophs, heterotrophic N_2_ fixation, aggregates, free-living cells, eutrophic river

## Abstract

Recent studies have indicated that heterotrophic diazotrophs are highly diverse and fix N_2_ in aquatic environments with *potentially* adverse conditions for diazotrophy, such as oxic and rich in total nitrogen. In this study, we compared the activity and diversity of heterotrophic diazotrophs associated with aggregates (>12 μm) to free-living cells in the eutrophic Qishon River during the winter and summer seasons. Overall, measured heterotrophic N_2_ fixation rates in the Qishon River ranged between 2.6–3.5 nmol N L^–1^ d^–1^. Heterotrophic N_2_ fixation was mainly associated with aggregates in the summer samples (74 ± 24%), whereas during the winter the bulk diazotrophic activity was mostly ascribed to the free-living fraction (90 ± 6%). In addition, immunolabeled micrographs indicated the presence of aggregate-associated heterotrophic diazotrophs in both seasons, while phototrophic diazotrophs were also captured during the winter. The richness of free-living and aggregate-associated heterotrophic diazotrophs were overall similar, yet the evenness of the later was significantly smaller, suggesting that few of the species gained advantage from particle lifestyle. The differences in the activity, micro-localization and diversity of the diazotrophic community were mostly attributed to spatiotemporal changes in the ambient *C*:*N* ratios (total organic carbon, TOC: total nitrogen) and the TOC concentrations. Taken together, our results shed new light on the contribution of heterotrophic diazotroph associated with aggregates to total heterotrophic N_2_ fixation in oxic, highly eutrophic aquatic environments.

## Introduction

Diazotrophs supply new bioavailable nitrogen compounds to primary producers in many marine and freshwater systems (Falkowski et al., 1998; Gruber and Galloway, 2008; Zehr and Capone, 2020). Recent reports indicate that the contribution of heterotrophic diazotrophs (bacteria and archaea) to new nitrogen (N) is more significant than previously estimated (Rahav et al., 2015; Bombar et al., 2016). High N_2_ fixation rates by heterotrophic diazotrophs were reported in various aquatic environments including estuaries, coastal water and aphotic zones in the open ocean (Hamersley et al., 2011; Rahav et al., 2013; Benavides et al., 2016). It was found that heterotrophic diazotrophs are widely distributed across the aquatic environments, from photic and aphotic layers of the open oceans to shallow coasts, estuaries, and lakes (Riemann et al., 2010; Moisander et al., 2017; Cornejo-castillo and Zehr, 2020). Heterotrophic diazotrophic representatives found in these environments have included various Proteobacteria, Firmicutes, Spirochetes, and Methanogens (Riemann et al., 2010; Farnelid et al., 2013; Bombar et al., 2016). Although limited information is currently available, heterotrophic diazotrophs were found to be hindered by high levels of dissolved inorganic nitrogen compounds (Klugkist and Haaker, 1984; Knutson et al., 2018), high concentrations of dissolved oxygen (Goldberg et al., 1987; Pedersen et al., 2018), as well as low availability of organic carbon (Rahav et al., 2016).

Previous studies suggested that heterotrophic diazotrophs associated with aggregates may gain metabolic advantages over free-living (planktonic) cells for several, not mutually exclusive, reasons: (1) there is a greater availability of labile carbon (C) sources after hydrolyzing the aggregates’ polysaccharide matrix (Bar-Zeev and Rahav, 2015). (2) Aggregates are often characterized by high *C*:*N* stoichiometry (Passow, 2002), thus imposing nitrogen limiting conditions on aggregate-associated bacteria. (3) Large-sized aggregates (few millimeters) may have low O_2_ micro-environments toward the center of the particle (Klawonn et al., 2015), thus minimizing the damage to the nitrogenase enzyme. Therefore, heterotrophic diazotrophs likely benefit from the association with aggregates in diverse environments considered adverse for N_2_ fixation, including oxygenated aphotic layers (Bonnet et al., 2013; Rahav et al., 2013; Benavides et al., 2016) and eutrophic estuaries and rivers with high dissolved nitrogen concentrations (Pedersen et al., 2018; Geisler et al., 2020). This idea was further reinforced by a recent modeling study showing the environmental thresholds of dissolved nitrogen and oxygen which preclude aggregate-associated heterotrophic N_2_ fixation in oceanic water (Chakraborty et al., 2021).

The Qishon River (South Eastern Mediterranean Sea) include an 11-km-long eutrophic estuary with high concentrations of NO_3_^–^ + NO_2_ (33.8–52.5 mg L^–1^) and PO^3–^_4_ (0.3–1.2 mg L^–1^) (Herut and Kress, 1997; Eliani-Russak et al., 2013; Bar-Zeev and Rahav, 2015), far exceeding the values reported for the Mediterranean’s coastal water (Drami et al., 2011; Kress et al., 2019). The high total nitrogen (TN) levels in the Qishon River should theoretically inhibit diazotrophy, and yet, significant heterotrophic N_2_ fixation rates were recently reported (0.4–5 nmol N L^–1^ d^–1^) (Geisler et al., 2020). Based on an immunolocalization approach, it was suggested that in such environments heterotrophic diazotrophs are associated and supported by aggregates. Nevertheless, high N_2_ fixation rates were not only found in the Qishon River, but also measured in other eutrophic environments, such as the Roskilde Fjord (Denmark), Jiaozhou Bay (China) and, Narragansett Bay (United States) (Bentzon-Tilia et al., 2014; Pedersen et al., 2018; Li et al., 2020; Hallstrøm et al., 2021). Although aggregates were suggested to support heterotrophic N_2_ fixation in environments with adverse conditions for diazotrophy (Bombar et al., 2016), the contribution of aggregate-associated diazotrophs to total heterotrophic N_2_ fixation is currently unknown.

In this study, we quantified the percent contribution of free-living and aggregate-associated heterotrophic diazotrophs to total N_2_ fixation in eutrophic environments. Specifically, N_2_ fixation rates and diversity of aggregate-associated vs. free-living heterotrophic diazotrophs were determined under varying ambient characteristics along the Qishon River. A complementary microscopic visualization of active diazotrophs was also carried out on aggregates collected across the eutrophic river.

## Materials and Methods

### Sampling Strategy

**FIGURE 1:**
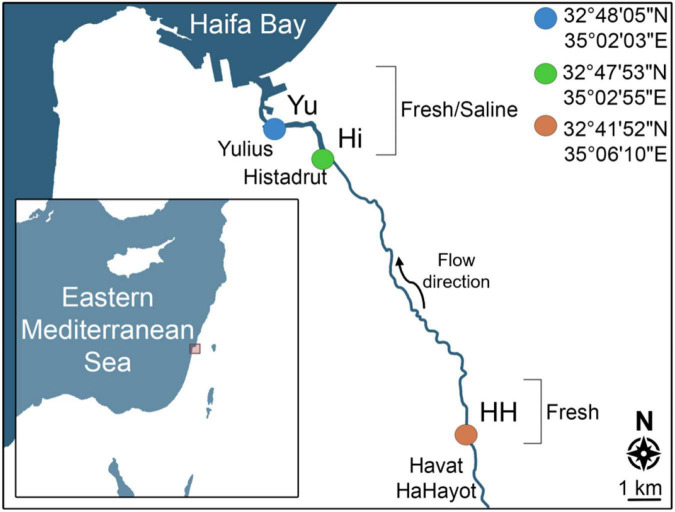
Map of the sampling locations along the Qishon River: upstream (Up, orange), mid-estuary (Mid, green), and estuary outflow (Out, blue), during winter (February 2018) and summer (July 2018).

Surface water (∼30 cm deep) was collected during the winter (February 2018) and summer (July 2018) months from three locations along the Qishon River (Table 1 and Figure 1): (i) an upstream, freshwater site (hereafter referred to as “Up”); (ii) the mid-point of the brackish estuary (”Mid”); and (iii) the estuary outflow (“Out”). The sampling locations were chosen based on a gradient of salinity and eutrophic conditions (Bar-Zeev and Rahav, 2015; Geisler et al., 2020). The following parameters were measured from the Qishon River water: salinity (EC-30, Phoenix Instrument), dissolved oxygen (DO, ProODO, YSI), pH (Cyberscan pH 11, Eutech), turbidity (Tu-2016, Lutron), heterotrophic diazotroph community structure and nitrogen fixation rates, as well as total organic carbon/nitrogen, and transparent exopolymer particles (specific details on the analytical methods are provided below).

**TABLE 1:** Spatiotemporal physicochemical properties of surface water from three sites along the Qishon River.

Station	Season	Temp. (°C)	Salinity (psu)	pH	Turbidity (NTU)	DO (% sat.)	TEP (mg GX L^–1^)	TOC (mg C L^–1^)	TN (mg N L^–1^)	*C*:*N* (mol: mol)
Upstream (Up)	Winter	11	0.5	7.7	544	67	9.5 ± 5.5	64.4 ± 11.2	18 ± 3	4.1
	Summer	28	3	8	77	62	0.5 ± 0.9	7.1 ± 0.4	12.9 ± 0.3	0.6
Mid-estuary (Mid)	Winter	12	0.3	7.5	1093	59	5.2 ± 2	26.8 ± 6.9	8.4 ± 2.2	3.8
	Summer	31	38	7.9	40	66	0.4 ± 0.02	5.4 ± 2.1	4.7 ± 0.8	1.3
Estuary outflow (Out)	Winter	12	0.7	7.5	822	60	4.3 ± 3.2	14.8 ± 4.4	4.1 ± 0.6	4.1
	Summer	31	37.7	8.2	26	100	0.7 ± 0.1	8.7 ± 3.4	4.4 ± 0.2	2.3

*Average and standard deviation were computed from three to four replicates for each sampling location per season.*

Water samples were collected and divided into three replicate bottles (1 L) in each season and sampling location. Bottles were pre-cleaned with 10% hydrochloride acid (HCl) and thoroughly washed with double-distilled water (DDW) to minimize external contamination. Each microcosm bottle was enriched with 5% of ^15^N_2_-enriched Qishon River water (*v:v*) (Mohr et al., 2010; Wilson et al., 2012). For more details on the^15^N_2_-enriched media preparation, see below. To focus on the role of heterotrophic diazotrophy rather than total N_2_ fixers (including photoautotrophs), the bottles were supplemented with 50 μM of the photosynthetic inhibitor 3-(3,4-dichlorophenyl)-1,1-dimethylurea (DCMU, final concentration, Sigma-Aldrich, D2425), and incubated for 48 h under dark conditions and ambient temperatures ranging from 25 to 27°C (Rahav et al., 2015, 2016; Benavides et al., 2018). The aggregates were continuously re-suspended by gentle shaking (100 rpm, TS-2620 Orbital Shaker, MRC, Israel). Subsamples from each bottle (0.4 L) were filtered after 48 h of incubation onto a 12-μm filter (Whatman WHA10400012), using a peristaltic pump (vacuum pressure lower than 300 mbar) to separate the aggregate-associated diazotrophs from the total heterotrophic N_2_ fixers. Collected aggregates were re-suspended in 20 ml of sterile Qishon water (filtrated 0.2 μm). The incubated water and aggregates subsamples were analyzed for N_2_ fixation rates (total heterotrophic and aggregate-associated diazotrophs), and immunolocalization of diazotrophs at the end of the incubation. Ambient water samples from each site were also analyzed for total organic carbon (TOC), total nitrogen (TN), and transparent exopolymer particles (TEP).

### Transparent Exopolymer Particles

Water samples (25–100 ml, 4 technical replicates for each sample) were filtrated using a vacuum (<150 mbar) onto 0.4-μm polycarbonate filters (GVS, Life Sciences) and stained with 5% Alcian Blue. TEP were extracted for 2 h in 5 ml of 80% sulfuric acid (diluted with DDW, H_2_SO_4_ 96%, Carlo Ebra, 410306). The absorption was measured in a spectrophotometer (Shimadzu GENESYTM) at a wavelength of 787 nm. TEP concentrations were calibrated with purified gum xanthan (GX, Sigma G1253) according to Passow and Alldredge (1995).

### Total Organic Carbon and Total Nitrogen

Water samples (*n* = 3, 15 ml each) were collected and stored in 20-ml glass tubes, acidified with 1 M of HCl (final concentration, pH 2.5) to remove dissolved inorganic carbon and kept at −20°C until analysis. Calibration was made according to a TOC/TN standards five-point calibration procedure (Kowalski et al., 2009) using TOC (Merck, 1090170100) and NH_4_^+^ (Merck, 1198120500) standards (from 0 to 100 mg L^–1^). TOC and TN were measured by a Multi N/C, Analytic-Jena, Germany with a detection limit of 0.3 mg L^–1^.

### N_2_ Fixation Rates

An artificial medium with similar salinity to the Qishon estuary was supplemented with a ^15^N_2_ gas tracer (99%, Cambridge Isotopes, lot #NLM-363-PK) at a 1:100 ratio (vol:vol) according to Mohr et al. (2010). Details on the artificial estuary water are provided in the Supplementary Material. The enriched water was vigorously shaken and stored at 4°C for ∼24 h to completely dissolve the ^15^N_2_ gas bubble. Note that the atom% was calculated rather than directly measured using membrane-introduction mass spectrometry (MIMS). Since the ^15^N_2_ bubble was completely dissolved, we assumed that the calculated atom% would be similar to the measured values. Moreover, the same ^15^N_2_ tank and ‘recipe’ were used to prepare the enriched estuary water in all stations and sampling campaigns. Therefore, even if the actual atom% values are skewed from the calculated ones (either underestimation or overestimation), the relative differences between seasons and stations could be establish.

The estuary water enriched with ^15^N_2_ was added to the microcosm bottles (1 L) bottles at 5% of the total volume, (Rahav et al., 2015) and the bottles were incubated for 48 h in the dark. At the conclusion of the incubation, the water samples were filtered onto pre-combusted glass fiber filters (GF/F,450°C, 4.5 h) and dried overnight in an oven at 65°C. Filters with a minimum 10 μg of particulate N (PN) per filter were used to resolve differences in N isotope ratios (White et al., 2020). The samples were analyzed with a CE Instruments NC2500 Elemental Analyzer (EA) and Thermo-Finnigan Delta Plus XP IRMS. Natural abundance (i.e., microcosms without the addition of ^15^N_2_) from each sampling location and season (*n* = 9) was subtracted from the corresponding samples. A standard curve of Acetanilide (C_8_H_9_NO) was generated before the measurements to determine the nitrogen mass of the samples (*R*^2^ > 0.99). The detection limit for ^15^N_2_ fixation was 0.02 nmole N L^–1^ d^–1^ which was a fold lower than the rates measured in this study, giving readability to the results.

### DNA Extraction and *nifH* Amplification

Diazotrophs associated with aggregates were collected from the ambient water on a 12-μm filter (Whatman, WHA10400012) by filtration of the Qishon waters (400 ml). Free-living diazotrophs were collected on a 0.4-μm polycarbonate filter from the filtered water (Millipore, HAWG047S6, 400 ml). Filters were kept in a lysis buffer (1 ml, additional information is provided in the Supplementary Material) and stored at −80°C. DNA was extracted from the samples by a phase separation method similar to that described in Bar-Zeev et al. (2008). Next, *nifH* genes were amplified using a nested polymerase chain reaction (PCR, Life Eco) process with *nifH* 3,4 and *nifH* 1,2 primers (Zehr and McReynolds, 1989; Gaby and Buckley, 2012). Sequencing was done on an Illumina MiSeq platform (RTL Genomics, Lubbock, TX, United States) according to Rahav et al., 2016. Additional information is provided in the Supplementary Material.

### Diazotroph Community Analysis

The *nif*H sequences were analyzed on a Quantitative Insights In to Microbial Ecology 2 pipeline (QIIME 2, version 2020.02) (Bokulich et al., 2018). The *nif*H sequences were de-multiplexed by associating a barcode with each sample (Marcel, 2011). Samples were de-noised using a DADA2 pipeline and aligned (Callahan et al., 2016). They were then grouped according to shared features, sampling location, and seasons to establish alpha diversity metrics and a principal coordinate analysis (PcoA). PCoA values (weighed UniFrac) were calculated using the q2-diversity script to measure quantitative dissimilarly between sequences (Lozupone et al., 2007). Primers were then extracted before classification (F – TGCGAYCCSAARGCBGACTC, R – ATSGCCATCATYTCRCCGGA). The taxonomic classifier (details in the Supplementary Material) was trained via Heller et al. (2014), and the sequences were assigned using the naive Bayes method (Pedregosa et al., 2011; Heller et al., 2014). Assigned sequences were visualized by taxonomic pie charts according to season, station, and filter size (0.4 and 12 μm). Unfortunately, a large fraction (54–98%) of the aggregate-associated heterotrophic diazotroph classes could not be assigned due to database constraints. *nif*H sequences files can be found in NCBI (Accession number: PRJNA735613).

### Immunolabeling Heterotrophic Diazotrophs Associated With Aggregates

Sample (50 ml) were filtered using gentle pressure (<150 mbar) through a 0.4-μm polycarbonate filter (GVS, Life Sciences, United States) at the end of the incubation, and stained for immunolabeling based on the protocol of Geisler et al. (2019). Briefly, filtered samples were fixed overnight with 5 ml of chilled ethanol before cells were permeabilized with 0.5% of dimethyl sulfoxide (1 ml, diluted with DMSO, Merck Millipore 102952) for 15 min at room temperature. Permeabilized cells on the filters were washed three times with 3 ml of phosphate buffer saline-Triton (PBST, 0.1% Triton X-100 in PBS, pH 7.2, Sigma-Aldrich). Diazotrophs were immunolabeled by tagging a primary antibody, anti-*nif*H (6 mg L^–1^ in 1 mg ml^–1^ PBS-bovine albumin serum), with the Mo-Fe nitrogenase enzyme (Agrisera Antibodies AS01 021A), which was then incubated for 1 h under dark conditions and washed following the above procedure. A secondary antibody that was conjugated to a fluorescein isothiocyanate (FTIC) fluorophore (6 μg ml^–1^, Thermo Fisher Scientific A-11039; Ex 495 nm, Em 519 nm) was added to the samples and incubated for 45 min under dark conditions. Samples were thoroughly washed with PBST (3×) to remove all the antibody traces and to minimize the non-bonded stain. In addition, TEP was stained with 4% Alcian blue solution for 10 s as described in Bar-Zeev et al. (2012) and washed with PBS. The polysaccharide matrix of the aggregates was identified by staining the same sample for 40 min with 200 μg ml^–1^ of Concanavalin-A (ConA, Thermo Fisher Scientific C11252; Ex 630 nm, Em 647 nm). Finally, the above sample was stained with 250 μg ml^–1^ of 4′,6-Diamidino-2-Phenylindole (DAPI, Thermo Fisher Scientific D1306; Ex 350 nm, Em 450 nm) for a 45-min incubation to visualize total bacterial cells. The filtered samples were washed with 5 ml of PBS and transferred to slides coated with poly-L lysine (1:1 with double-distilled water, Sigma-Aldrich, P8290), protected with a cover slip (2 cm × 1 cm), and sealed to minimize complete dehydration. The stained samples were imaged by a confocal laser scanning microscope (CLSM, Zeiss, LSM880) equipped with 405, 488, 561, and 633 nm lasers. Aggregates were located in the bright field mode by identifying the TEP matrix. Aggregates were captured with 2.5× (EC Plan-Neofluar 2.5×/0.085 M27) or 10× (10×/0.45 M27) lenses, and specific locations were captured with a 63× lens (Plan-Apochromat 63×/1.4 Oil DIC M27). Negative control samples, namely without the anti-*nif*H antibody were captured to determine unspecific tagging and/or autofluorescence. The settings (gain and laser intensity) of non-stained samples were applied and adjusted to the rest of the samples. CLSM images were analyzed using the ZEN blue edition (Zeiss).

### Statistical Analyses

Normality distribution of the data was first validated using the Shapiro–Wilk test. Differences in TOC, TN, *C*:*N*, and TEP between seasons were analyzed using a student *t*-test (*n* = 6–12). Differences in N_2_ fixation between sampling locations, seasons (summer and winter), and lifestyle (free living and aggregates associated), were analyzed using a one-way ANOVA and Fisher-LSD means comparison (*n* = 9). Free-living and aggregate-associated N_2_ fixation were correlated with TOC, TN, and *C*:*N* using a Pearson correlation test. All calculations were done using the XLSTAT 2019 software (Addinsoft, New York, NY, United States) with a confidence level of 95% (α = 0.05).

## Results and Discussion

### The Hyper-Eutrophic Characteristics of the Qishon River

The physicochemical characteristics of the Qishon River were markedly different for most parameters between the winter and the summer as well as between the three locations along the river (Table 1). The Qishon River was overall oxygenated with O_2_ saturation higher than 59%. The reduction in O_2_ saturation was likely resulted from intense microbial activity previously reported for eutrophic environments such as the Qishon River (Bar-Zeev and Rahav, 2015). During the winter, salinity was low throughout the river (0.3–0.7 psu), due to frequent precipitation events (∼700 mm per year), resulting in high freshwater fluxes^1^. During the summer, salinity in the upstream location was slightly brackish (3 psu), while much higher in the estuary stations (midstream and downstream, ∼38 psu) due to seawater penetration inland (39–40 psu), as often occur in estuaries (Eliani-Russak et al., 2013). In addition, the Qishon water was more turbid during the winter (544–1093 NTU) than the summer (26–77 NTU), with significant differences between stations (ANOVA, *p* < 0.05, *n* = 6). Higher turbidity during the winter was likely resulted from enhanced sediment suspension, increasing nutrient concentrations in the overlying water. TOC declined from the upstream to the outflow stations during the winter (5–64 mg C L^–1^, ANOVA, *p* < 0.005, *n* = 9), whereas significantly lower values without a clear spatial trend were measured in the summer (5–9 mg L^–1^, *t*-test, *p* < 0.001). TN also exhibited a marked decrease from the upstream location to the river outflow during the winter (from 18 to 4 mg N L^–1^), while a weaker decline was found in the summer (from 13 to 4 mg L^–1^). The high TOC and TN concentrations, especially during the winter, were most likely due to intense agricultural runoff into the Qishon River catchment area. The nutrient measurements indicate that the Qishon River is a hyper-eutrophic (TN > 1 mg N L^–1^) environment according to the United States National Oceanic and Atmospheric Administration (NOAA, 1996). Corresponding *C*:*N* ratios (mol:mol) were 1.5- to 7-fold higher during the winter (3.4 ± 0.2) than the summer (1.2 ± 0.8), with no apparent differences along the river (Table 1). Nonetheless, these *C*:*N* ratios were much lower than the typical Redfield ratio of 6.6:1 (Redfield, 1934). Therefore, the differences in the *C*:*N* ratio between summer and winter were mostly attributed to higher concentrations of TOC measured in the winter (Table 1). TEP concentrations in the Qishon River ranged from 0.4–9.5 mg GX L^–1^ and were much higher than those usually reported from the nearby SE Mediterranean Sea (Bar-Zeev et al., 2011). During the winter, TEP values were ∼twofold higher at the upstream station than at the estuary outflow (Table 1). In contrast, no differences in TEP concentrations were measured along the Qishon River during the summer (Table 1). TEP, as part of the particulate organic pool, also play a central role in aggregation, forming the polymeric matrix of aquatic aggregates (Wurl et al., 2011; Bar-Zeev and Rahav, 2015; Turner, 2015). Therefore, high TEP concentrations are a fair indication of large numbers of aggregates (Bar-Zeev and Rahav, 2015). These type of aggregates were previously suggested to support diazotrophic activity, especially in environments with adverse conditions for diazotrophy such as the oxygenated (O_2_ saturation >59%), TN-rich water found in the Qishon River (Bonnet et al., 2013; Rahav et al., 2013; Geisler et al., 2020).

### Free-Living vs. Aggregate-Associated Heterotrophic N_2_ Fixation

Overall, the total heterotrophic N_2_ fixation rates throughout in the Qishon River (i.e., free-living + aggregate-associated) were an order of magnitude higher than in neighboring seas (Benavides et al., 2015; Rahav et al., 2015), yet in agreement with other estuary environments (Supplementary Table 1). Total N_2_ fixation rates by heterotrophic diazotrophs were different between winter (3.5 ± 1.9 nmol N L^–1^ d^–1^) and summer (2.6 ± 0.8 nmol N L^–1^ d^–1^) along the eutrophic Qishon River (*t*-test, *p* < 0.01). During the winter, total heterotrophic N_2_ fixation was significantly greater upstream (5.3 ± 1.9 nmol N L^–1^ d^–1^) than in the estuary stations (2.6 ± 1.7 nmol N L^–1^ d^–1^). In contrast, the total N_2_ fixation rates measured along the Qishon River were similar during the summer (2.3–3.1 nmol N L^–1^ d^–1^).

**FIGURE 2:**
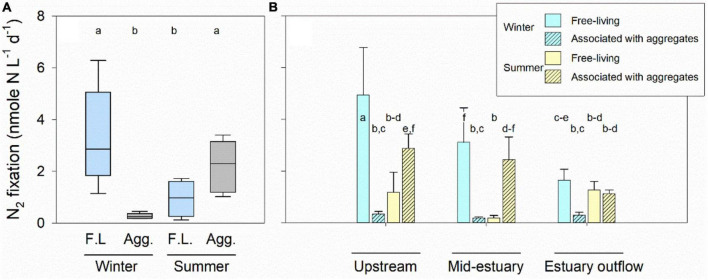
Spatiotemporal changes in heterotrophic N_2_ fixation along the Qishon River. N_2_ fixation rates by free-living (F.L., blue) and aggregate-associated (gray) diazotrophs were averaged from all the sampling locations in winter and summer **(A)**. Spatial N_2_ fixation represent the rates measured from each sampling location in both seasons **(B)**. The letters above the boxplots (*n* = 9) and bars (*n* = 3) represent the statistical difference between samples (ANOVA, Fisher LSD *post hoc* test, *P* < 0.05).

N_2_ fixation rates by free-living heterotrophic diazotrophs during the winter were ∼3.4 times higher than in the summer (Figure 2A). Concurrently, N_2_ fixation by free-living heterotrophs was ∼11 times greater than associated with aggregates, accounting for ∼90% of the total heterotrophic N_2_ fixation during the winter (Table 2). In addition, a gradual decrease in heterotrophic N_2_ fixation by free-living diazotrophs was measured from the upstream location and toward the estuary outflow (Figure 2B). During the summer, however, N_2_ fixation by aggregate-associated heterotrophic diazotrophs was 2.3 times higher than that by the free-living fraction (Figure 2A). In addition, a gradual reduction in N_2_ fixation by aggregate-associated heterotrophic diazotrophs was measured toward the estuary outflow, while no trend was found for the free-living fraction (Figure 2B). More importantly, the average contribution of aggregate-associated heterotrophic diazotrophs to total heterotrophic N_2_ fixation during the summer was 74% (Table 2).

**TABLE 2:** Summary of the percent (mean) contribution of aggregate-associated heterotrophic diazotrophs to total heterotrophic N_2_ fixation (defined as 100%).

Station	Winter (%)	Summer (%)
Upstream	7 ± 1	80 ± 22
Mid-estuary	7 ± 2	94 ± 5
Estuary outflow	16 ± 8	47 ± 6
Average% contribution	10 ± 6	74 ± 24

*The mean and standard deviation was calculated from three replicates.*

**FIGURE 3:**
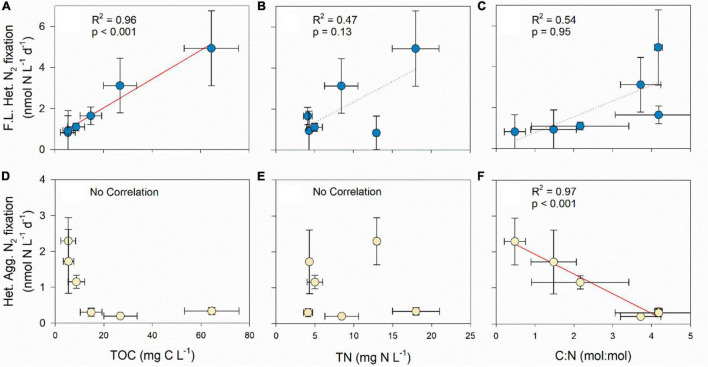
Pearson correlation test between free-living (F.L.) and aggregate-associated (Agg.) heterotrophic (het.) N_2_ fixation to TOC **(A,D)**, TN **(B,E)**, and *C*:*N* **(C,F)**. The statistical results are shown in the different panels (*n* = 6). Solid trend line indicates that the correlation between the different variables was significant, while dash line shows the trend only.

A positive and significant correlation was found between N_2_ fixation by free-living heterotrophic diazotrophs and TOC (Figure 3A). In addition, a positive trend was found between TN concentrations and the *C*:*N* ratio to N_2_ fixation by free living diazotrophs, yet these relationships were non-significant (Figures 3B,C). N_2_ fixation rates by free-living heterotrophic diazotrophs were previously shown to be highly dependent on the availability of organic carbon (Rahav et al., 2016). It can be inferred that even in eutrophic environments such as the Qishon River, free-living heterotrophic diazotrophs can be limited by carbon availability if the *C*:*N* ratio is lower than the ‘typical’ ∼6.6:1 Redfield ratio.

During the winter, frequent runoffs of organic matter (measured as TOC) from agricultural sources (e.g., cattle farming) and sewage from wastewater treatment plants located upstream have likely supported the activity of free-living heterotrophic N_2_ fixation (Figure 2A and Table 2). It should be noted that significant fraction of the measured TOC was likely labile as previously reported for organic material discharged to the aquatic environment from these type of anthropogenic sources (Worrall et al., 2019; Park et al., 2021). In contrast to organic carbon, it was previously shown that high concentrations of dissolved inorganic nitrogen in the surrounding water may inhibit natural diazotrophic populations (Capone et al., 1990; Mulholland and Capone, 2001; Fu and Bell, 2003) as well as cyanobacterial diazotrophs (Milligan et al., 2007; Dekaezemacker and Bonnet, 2011; Knapp et al., 2012) and very little is currently known about heterotrophic diazotrophs (either free-living or aggregate associated). Nevertheless, the high TN concentrations measured in the Qishon River during the winter should have, theoretically, hinder N_2_ fixation by free-living heterotrophic diazotrophy. It can be surmised that discharge of organic carbon from anthropogenic sources and the relatively low *C*:*N* ratio (compared to the Redfield ratio) during the winter led to high N_2_ fixation rates by free-living heterotrophic diazotrophs.

During summertime, when the ambient TOC concentrations throughout the Qishon River were lower than the winter, heterotrophic diazotrophic activity were mostly dependent on the association with aggregates (Table 1). It was found that N_2_ fixation by heterotrophic diazotrophs that colonize aggregates was not linearly correlated to TOC measured in the surrounding water (Figure 3D). Similarly to previous studies, it is highly likely that these communities gained their carbon source by hydrolyzing the aggregate matrix rather than from the surrounding water (Bar-Zeev and Rahav, 2015; Datta et al., 2016; Bižic-Ionescu et al., 2018). It is also possible that the fraction of labile carbon during the summer was smaller (than the winter), as no reports of significant anthropogenic runoff into the Qishon River (e.g., sewage) were known or published. Moreover, the *C*:*N* ratio of aggregates is often higher than in the aquatic environment, namely >6.6:1 (Engel and Passow, 2001). Thus, bacteria that colonize these aggregates will be limited by low DIN concentrations, providing advantage to heterotrophic diazotrophs (Figure 3E). Nonetheless, a negative and significant relationship was found between the *C*:*N* ratio and aggregate-associated heterotrophic N_2_ fixation (Figure 3F). This negative correlation suggests that the advantage gained by heterotrophic diazotrophs that colonized aggregates had lower significance if the *C*:*N* ratio increases toward the Redfield ratio, even in the oxygenated Qishon River (O_2_ saturation > 60%). From a different perspective, it can be inferred that free-living heterotrophic diazotrophs may actively fix N_2_ under these conditions (oxygenated water with high TN concentrations) if the availability of organic carbon can support intense catabolism to reduce intracellular O_2_ concentrations (Inomura et al., 2018).

### Community Structure of Free-Living and Aggregate-Associated Diazotrophs

**FIGURE 4:**
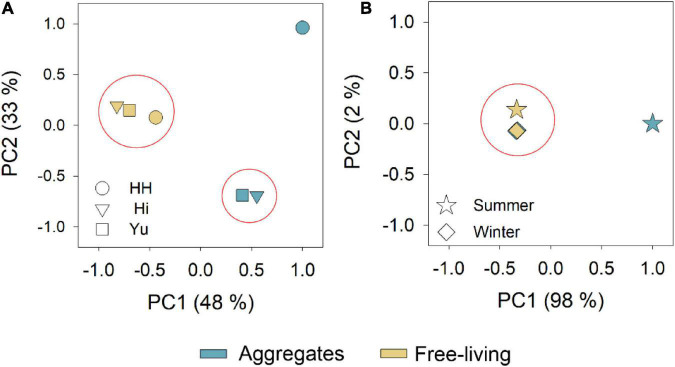
Principal coordinate analysis (PCoA) plots showing the distance in bacterial diversity (derived from *nif*H analyses) using weighted UnifFrac at the different sampling locations **(A)** and sampling seasons **(B)**. Colors represent free-living (yellow) and aggregate-associate (blue) fractions. The shapes of the symbols in the figure **(A)** indicate the different sampling locations; upstream (circle), mid-estuary (triangle) and estuary outflow (square). Shapes of the symbols in figure **(B)** indicate the summer (star) and winter (diamond).

Shannon index of the diazotrophic communities (regardless to the sampling season or location) indicated that the richness of the diazotrophic community was similar between the aggregate-associated and free-living fractions (Kruskal–Wallis test *p* > 0.05). Differently, the evenness of the free-living fraction was significantly greater than that of the diazotrophic communities associated with aggregates (Kruskal–Wallis test, *p* < 0.05). In addition, PCoA analyses indicated that free-living diazotrophs clustered together, regardless of the sampling location. However, diazotrophs associated with aggregates in the stream differed from those found in the estuary (Figure 4A). During the summer, aggregate-associated diazotrophs differed from the other fractions (Figure 4B), although statistical tests by analysis of similarities and permutational analysis of variance could not be concluded.

Following the above, it can be deduced that diazotroph diversity found in this eutrophic environment was similar between free-living and aggregate associated. However, the lower evenness values of diazotrophs associated with aggregates compared to the free-living fraction may indicate that few of these species gained specific benefits after colonizing the particles (Hollibaugh et al., 2000; Zhang et al., 2012). These particle associated communities are known to gain metabolic advantages by a wide spectrum of extracellular enzymes that efficiently biodegrade the aggregate organic matrix (Grossart et al., 2006; Ghiglione et al., 2007).

**FIGURE 5:**
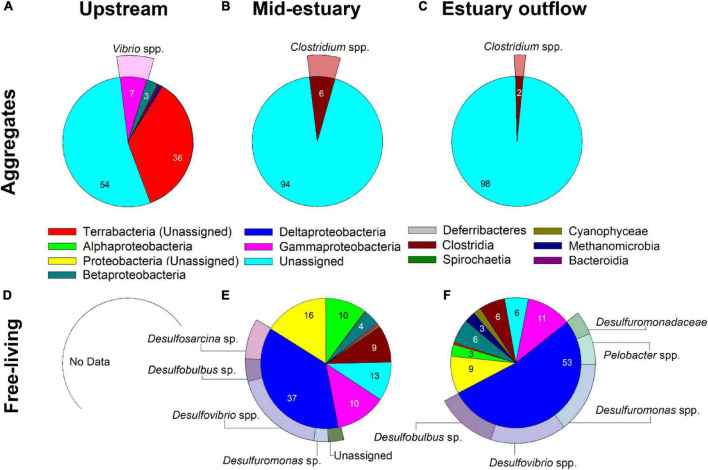
Summertime diazotrophic community composition at the Qishon River derived from *nifH* analyses associated with aggregates **(A–C)** and as free-living bacteria **(D–F)**. Numbers represent relative abundance (%, mean = 3,700 OTUs) of the (class level) OTUs in the sample. Peripheral-pie slices represent the genus of the most abundant (and assigned) class in a sample. The remaining classes are shown in Supplementary Table 2. Data for free-living diazotrophs upstream is not available due to technical constrains.

During the summer, Terrabacteria accounted for 36% of the identified OTUs, while beta, gamma and deltaproteobacteria together constituted 11% of the aggregate-associated diazotroph classes upstream (Figure 5A). Differently, the diazotroph class that could be assigned to aggregates at the Qishon estuary was Clostridia (Figures 5B,C). The main genus that could be identified in the upstream station was *Vibrio* spp., whereas *Clostridium* spp. was found only in the mid-estuary and outflow stations. *Vibrio* spp. are heterotrophic, facultative anaerobes, often found associated with aggregates (Dang and Lovell, 2016; Lovell, 2017), and are ubiquitous in aquatic environments ranging from fresh to saline water (Criminger et al., 2007; Lyons et al., 2010; Lovell, 2017; Geisler et al., 2019). Several *Vibrio* species, such as *Vibrio diazotrophicus*, *Vibrio natriegens*, and *Vibrio parahaemolyticus*, were reported to fix N_2_ under anaerobic and aerobic conditions (Guerinot et al., 1982; Coyer et al., 1996; Geisler et al., 2019). *Clostridium* spp. are heterotrophic, anaerobic bacteria with numerous nitrogen-fixing species (Palacios and Newton, 2005). We suggest that these heterotrophic bacterial diazotrophs would proliferate on particles by exploiting the micro-anaerobic environments found in the aggregate as previously suggested by Mestre et al. (2017).

Free-living diazotrophs during the summer comprised mostly alpha, gamma and deltaproteobacteria (37–53%) in the estuary locations, yet no data was retrieved from the upstream location (Figure 5D). The *Desulfovibrio* genus constituted from 29–50% of the total deltaproteobacteria class. In addition, *Desulfobulbus* sp. and *Desulfuromonas* sp. were also abundant, accounting for 50% of the total diazotrophic reads (Figures 5E,F). These genera are all sulfate-reducing, aerotolerant anaerobes that were found to fix N_2_ in various aquatic environments such as seagrass meadows (Welsh, 2000), as well as coastal and estuarine sediments (Thajudeen et al., 2017; Jabir et al., 2021). We surmise that these sulfate-reducing benthic diazotrophs were re-suspended from the sediment, as the water level in the Qishon estuary was low in the summer (∼1–2 m). Previous studies have also reported that sediment resuspension processes can affect water column’s diazotrophic communities in shallow water (Pedersen et al., 2018; Zilius et al., 2021). We hypothesize that these genera could fix N_2_ as free-living cells by hydrolyzing the high concentrations of organic material found in the Qishon River.

**FIGURE 6:**
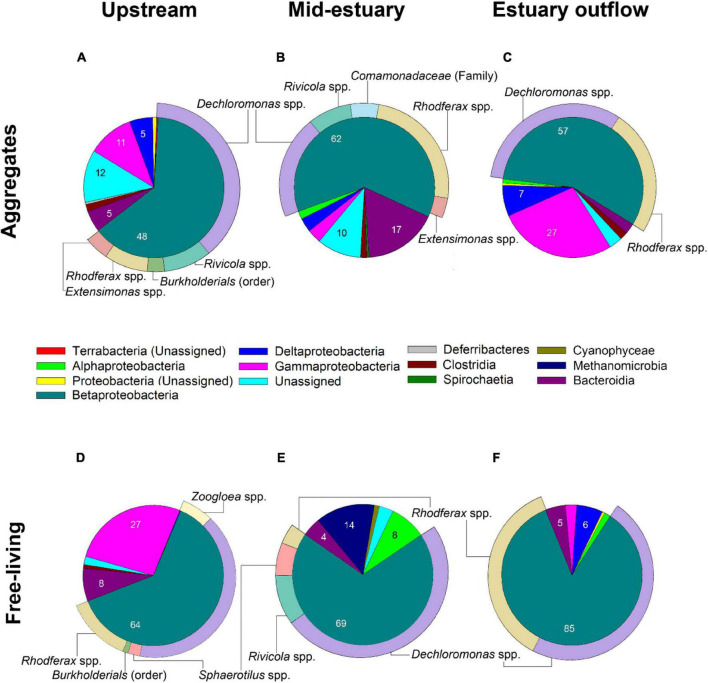
Wintertime diazotrophic community composition at the Qishon River derived from *nif*H analyses associated with aggregates **(A–C)** and as free-living bacteria **(D–F)**. Numbers represent relative abundance (%, mean = 5,300 OTUs) of class level OTUs in the sample. Sub-pie slices represent the genus of the most abundant (and assigned) class in a sample. The remaining classes are shown in Supplementary Table 2.

During the winter, the dominant classes of both free-living and aggregate-associated diazotrophs were mainly betaproteobacteria (48–85%) and gammaproteobacteria (3–27%). Specifically, *Dechloromonas* sp. (32–71%), and *Rhodoferax* sp. (5–43%) were the dominant genera in both fractions (Figure 6). *Dechloromonas* sp. are facultative anaerobic diazotrophs, often found in eutrophic environments (Cai et al., 2014). *Dechloromonas* sp. could fix N_2_ as free-living cells (Jabir et al., 2020), or after they colonize the aggregates (Cai et al., 2014; Farnelid et al., 2019). *Rhodoferax* spp. genera were reported to have optimal growth from 12–18°C (similar to the temperatures found in the Qishon River during the winter) and diverse metabolic pathways ranging from phototrophic carbon fixation to aerobic and anaerobic catabolism (Hiraishi et al., 1991). The *Rhodoferax* spp. were shown to grow on different nitrogen sources or fix N_2_ under various conditions (Madigan et al., 2000; Baker et al., 2017). Our results suggest that *Rhodoferax* spp. may switch between phototrophic and heterotrophic N_2_ fixation, following exposure to the photosynthetic inhibitor (DCMU) and the dark incubations carried in the reported study.

### Micro-Localization of Diazotrophs on Aggregates Along the Qishon River

**FIGURE 7:**
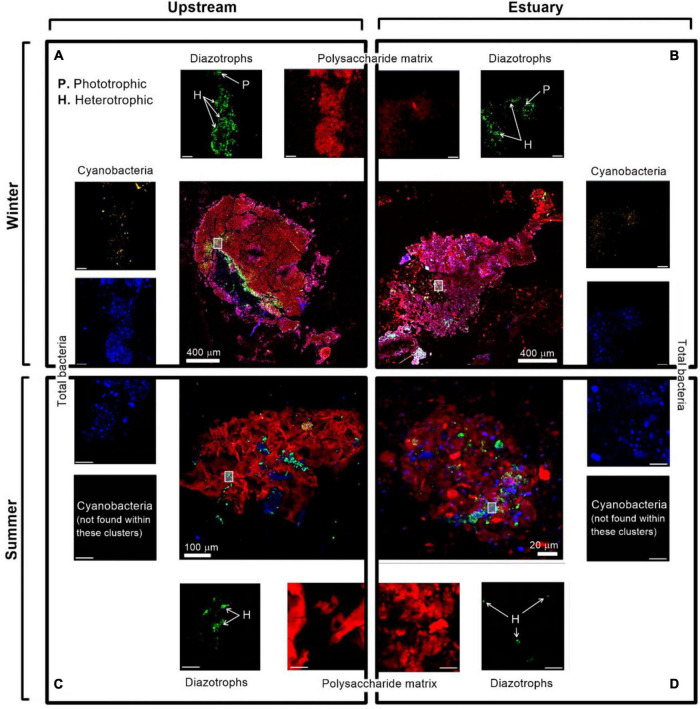
Immunolabeling micrograph images of diazotrophs associated with aggregates collected from the Qishon River (**A,C** - stream; **B,D** - Estuary) during the winter (top panels) and summer time (bottom panels). Images were captured by a confocal laser scanning microscope with a double close-up of bacteria associated with aggregates. Images were taken in four layers (sub-plots around each aggregate) as cyanobacteria by autofluorescence of the phycoerythrin pigment (orange/white), diazotrophs by immunolabeling of nitrogenase [green, bacteria by DAPI (blue), and polysaccharides by ConA (red). The mark on the aggregates represents a close-up of the image. The reported scale bar in the top inserts is 10 μm, while the scale bar in the bottom inserts is 5 μm. H refers to heterotrophic diazotrophs and P to phototrophic diazotrophs, distinguished by the phycoerythrin pigment]. Additional microscopy images are provided in the Supplementary Figure 1.

Large-sized aggregates (>500 μm, *n* = 40) comprising polysaccharides (stained by ConA) and dense microbial clusters (tagged with DAPI) were often found in the winter along the Qishon River (Figures 7A,B). In contrast, smaller aggregates (<500 μm, *n* = 23) were mostly found during the summer sampling (Figures 7C,D). Diazotrophs were often captured on these aggregates by immunolabeling the nitrogenase enzyme with fluorescent antibodies (Figure 7 and Supplementary Figure 1), suggesting they are actively fixing N_2_. Diazotrophs associated with aggregates were found, regardless of the season or sampling location. Yet, during the wintertime, diazotrophic clusters were often larger (50–500 μm) and included both heterotrophic and unicellular phototrophic diazotrophs representatives (Figures 7A,B, green and orange inserts). Differently, during the summertime, most of the captured diazotrophs associated with aggregates were heterotrophs (Figures 7C,D, green inserts).

Aggregates were previously suggested to support heterotrophic diazotrophs, especially in environments characterized by adverse conditions for diazotrophy (Bombar et al., 2016; Rahav et al., 2016). The surface water of the Qishon River can be considered as such due to the oxygenated, low *C*:*N* ratio conditions, particularly during the summer months (Table 1) (Bar-Zeev and Rahav, 2015). These conditions should theoretically impair free-living diazotrophs and prioritize the activity of aggregate colonizers (Figure 7). It should be noted that it was surprising to capture active phototrophic diazotrophs associated with aggregates (Figure 7), especially after prolonged dark incubation with a photosynthetic inhibitor. Based on the diversity analyses, cyanobacteria were hardly detected in our samples during the winter (Figure 6). However, it was previously shown that many phototrophic diazotrophs, such as the *Rhodoferax* sp. found in our aggregate samples (Figures 7C,D), have mixotrophic capabilities, namely the ability to switch and metabolize available carbon if needed (Jin et al., 2020). Therefore, it is possible that these unicellular phototrophic diazotrophs were not actively fixing carbon via photosynthesis, but rather metabolizing available carbon by oxic respiration. Why these mixotrophic diazotrophs were not colonizing aggregates during the summer (Figures 6, 7C,D) remains to be elucidated.

In addition to the above, heterotrophic diazotrophs were expected to be located toward the center of the aggregate, where lower oxygen concentrations are more likely to be found (Klawonn et al., 2015). Oxygen concentrations within the aggregate depend on: (i) saturation of dissolved O_2_ in the ambient water (Paerl and Prufert, 1987); (ii) diffusion rate of O_2_ (Ploug and Passow, 2007); (iii) oxygen consumption rate by the aggregate-colonizing microbes (Klawonn et al., 2015); and (iv) the aggregate size (Klawonn et al., 2015). The aggregates found in the Qishon River, compared to those reported in model experiments were much smaller (a few hundreds of microns μm vs. >3 mm, Klawonn et al., 2015). Therefore, it is unlikely that an anoxic gradient could develop and that heterotrophic diazotrophs would be present toward the center of the aggregates, as dissolved O_2_ would diffuse throughout the particle. Nonetheless, it is possible that micro-environments of reduced oxygen concentrations could be found in proximity to the bacterial clusters seen on these aggregates (not necessarily diazotrophs, Figure 7, blue inserts) due to enhanced respiration rates, thus indirectly supporting diazotrophic activity.

## Conclusion

*The Qishon River is characterized by high nitrogen concentrations, low C:N ratio, and oxygenated water, and yet, significant N_2_ fixation rates were measured throughout this eutrophic environment, in both the summer and winter seasons*. We found that during the summer, when the *C*:*N* ratio was exceptionally low (<1), heterotrophic N_2_ fixation associated with aggregates accounted for ∼74% of the total rates measured. In contrast, during the winter, N_2_ fixation by aggregate-associated heterotrophic diazotrophs was low (<10%), likely because TOC concentrations and the *C*:*N* ratio were much higher. Currently, the number of aggregates per liter and the abundance of diazotrophs that colonize each particle are unknown. Such information will enable quantifying the specific contribution of heterotrophic diazotrophs to the new nitrogen in a given environment, including the eutrophic Qishon River.

Taken together the above, results of this study indicate that nitrogen fixation rates by aggregate associated heterotrophic diazotrophs cannot be neglected in the eutrophic Qishon River. Moreover, we suggest that significant contribution of aggregate-associate heterotrophic diazotrophs to total N_2_ fixation will not be restricted to the Qishon River only but can be found to be central in other marine and freshwater environments with adverse conditions for diazotrophy.

## Data Availability Statement

The original contributions presented in the study are included in the article/Supplementary Material, further inquiries can be directed to the corresponding authors.

## Author Contributions

EB-Z, EG, and ER conceived and designed the experiments, analyzed the data, and wrote the manuscript. EG performed the samplings. All authors contributed to the article and approved the submitted version.

## Conflict of Interest

The authors declare that the research was conducted in the absence of any commercial or financial relationships that could be construed as a potential conflict of interest.

## Publisher’s Note

All claims expressed in this article are solely those of the authors and do not necessarily represent those of their affiliated organizations, or those of the publisher, the editors and the reviewers. Any product that may be evaluated in this article, or claim that may be made by its manufacturer, is not guaranteed or endorsed by the publisher.

## Footnotes

1
https://ims.data.gov.il/
