# Supplementary material for: Contribution of Heterotrophic Diazotrophs to N2 Fixation in a Eutrophic River: Free-Living vs. Aggregate-Associated
Source: Front Microbiol. 2022 Feb 14;13:779820. doi: 10.3389/fmicb.2022.779820 (PMC8882987; doi:10.3389/fmicb.2022.779820)
Supplement: Supplementary file 1 [file Data_Sheet_1.docx]

**Supporting Information**

**Contribution of Heterotrophic Diazotrophs to N_2_ Fixation in a Eutrophic River: Free-Living vs. Aggregate-Associated**

Eyal Geisler^1,2^, Eyal Rahav^2^*, & Edo Bar-Zeev^1^*

^1^Zuckerberg Institute for Water Research, Jacob Blaustein Institutes for Desert Research, Ben-Gurion University of the Negev, Sede Boqer Campus, 84990, Israel.
^2^Israel Oceanographic and Limnological Research, National Institute of Oceanography, Haifa, 31080, Israel.

*Corresponding Authors: [eyalrahav@ocean.org.il](mailto:eyalrahav@ocean.org.il) ; [barzeeve@bgu.ac.il](mailto:barzeeve@bgu.ac.il)

**Materials and Methods**

Preparation of *^15^N_2_-enriched artificial medium with similar salinity to the Qishon estuary*: Sea salts (Advanced Pro Formula Salt, Royal Nature) were dissolved in double-distilled water (15 g L^-1^) and filtrated through a coarse filter paper (Macherey-Nagel). Sodium bicarbonate, NaHCO_3_, was added (15 mg L-1) to maintain a pH of 7–7.2. Vitamins (0.75 µM cobalamin, 4 µM biotin, and 0.8 µM thiamine HCl) and trace metals (4.8 mM [FeCl_3_]x6H_2_O, 17 µM Na2EDTAx2H_2_O, 40 µM [CuSO_4_]x5H_2_O, 28 µM [NaMoO_4_]x2H_2_O, 76 µM [ZnSO_4_]x7H_2_O, 42 µM [CoCl_2_]x6H_2_O, and 1 mM [MnCl_2_]x4H_2_O) solutions were filtered through a 0.22-µm filter (Millex SLGV033RS) and added to the sterile media. A bottle of estuary water was filled until there was no headspace and enriched with an ^15^N_2_ gas tracer (99%, Cambridge Isotopes, lot #NLM-363-PK) at a 1:100 ratio (vol:vol).

*DNA extraction and nif*H *amplification*: Filtered samples were kept with 1 ml of lysis buffer (40 mM EDTA, 50 mM Tris HCl pH = 8.3, 0.75 M Sucrose) at -80 °C until the DNA extraction. The *nif*H genes were amplified using Takara Taq (R011, Takara, Japan) and primers (Hylabs, Israel) in a polymerase chain reaction (PCR, Life Eco, Bioer Technology, China) in two stages. The first stage was done with r: TTYTAYGGNAARGGNGG and f: ATRTTRTTNGCNGCRTA under the following conditions: 94 °C for 5 min, 30 cycles of 94 °C for 1 min, 50 °C for 1 min, 72 °C for 1 min, and a final extension at 72 °C for 1 min. The second stage was done with r: ADNGCCATCATYTCNCC and f: TGYGAYCCNAARGCNGA under the following conditions: 94 °C for 5 min, 30 cycles of 94 °C for 1 min, 57 °C for 1 min, 72 °C for 1 min, and a final extension at 72 °C for 1 min. PCR products were visualized using gel electrophoresis, and they were validated with a positive control (*V. natriegens*) and a negative control (double-distilled water, DDW).

*Diazotroph structure analysis, database:* Sequence primers were extracted before the classification (f: TGCGAYCCSAARGCBGACTC, r: ADNGCCATCATYTCNCC). The taxonomic classifier was trained via the *nif*H database from Heller at al., 2014 (downloaded from <https://www.jzehrlab.com/>) (Heller et al., 2014). The nifH sequences were classified using a taxonomy database (NCBI) to build a taxonomic table. OTUs were assigned using the naive Bayes method and were visualized by a taxonomic bar plot according to season, station, and filter size (0.4 and 12 µm).

**Results and Discussion**

**Table S1.** Statistical measurements for comparing variables. The table comprise normality measurements, chosen statistical method, number of observations (n) and results (p-value)

**Table S1**. N_2_ fixation rates by heterotrophic bacteria and dominant diazotrophs in corresponding studies.

| **Location** | **N_2_ fixation (nmole N L^-1^ d^-1^)** | **Dominated classes** | **Nutrient status** | **Reference** |
| --- | --- | --- | --- | --- |
| Qishon River, Israel | 1-3 (Free Living) | Beta and Deltaproteobacteria | Eutrophic | This study |
|  | 0.3-2 (Aggregates) | Unassigned |  |  |
| Gulf of Aqaba, Israel | 0.02-0.38 | N.A | Oligotrophic | (Rahav et al., 2013) |
| Levantine Basin, Israel | 0.01-0.24 | Alpha and Gammaproteobacteria |  |  |
| South Pacific Ocean | 0.26 | Alpha, Gamma and Deltaproteobacteria | Oligotrophic | (Bonnet et al., 2013) |
| Southeastern Mediterranean Sea, Israel | 0.10-0.15 | Alpha-Gammaproteobacteria | Oligotrophic | (Rahav et al., 2016) |
| Roskilde Fjord, Denmark | ~3 (Bulk) | Alpha-Deltaproteobacteria | Eutrophic | (Pedersen et al., 2018) |
|  | ~2 (Filtrate) | Unassigned |  |  |
| New Caledonia | ~0.1 | Gammaproteobacteria | Oligotrophic | (Benavides et al., 2018) |
| Jiaozhou Bay, China | ~4.8 | Deltaproteobacteria | Eutrophic | (Li et al., 2020) |
| Cochin Estuary, India | 2.4-48 | Alpha-Deltaproteobacteria and Cyanobacteria | Eutrophic | (Jabir et al., 2020) |
| Narragansett Bay, United States of America | 0.08-9.41 | Deltaproteoacteria | Eutrophic | (Hallstrøm et al., 2021) |

**Table S2**. Representative OTU classes (%) according to sampling location and seasons.


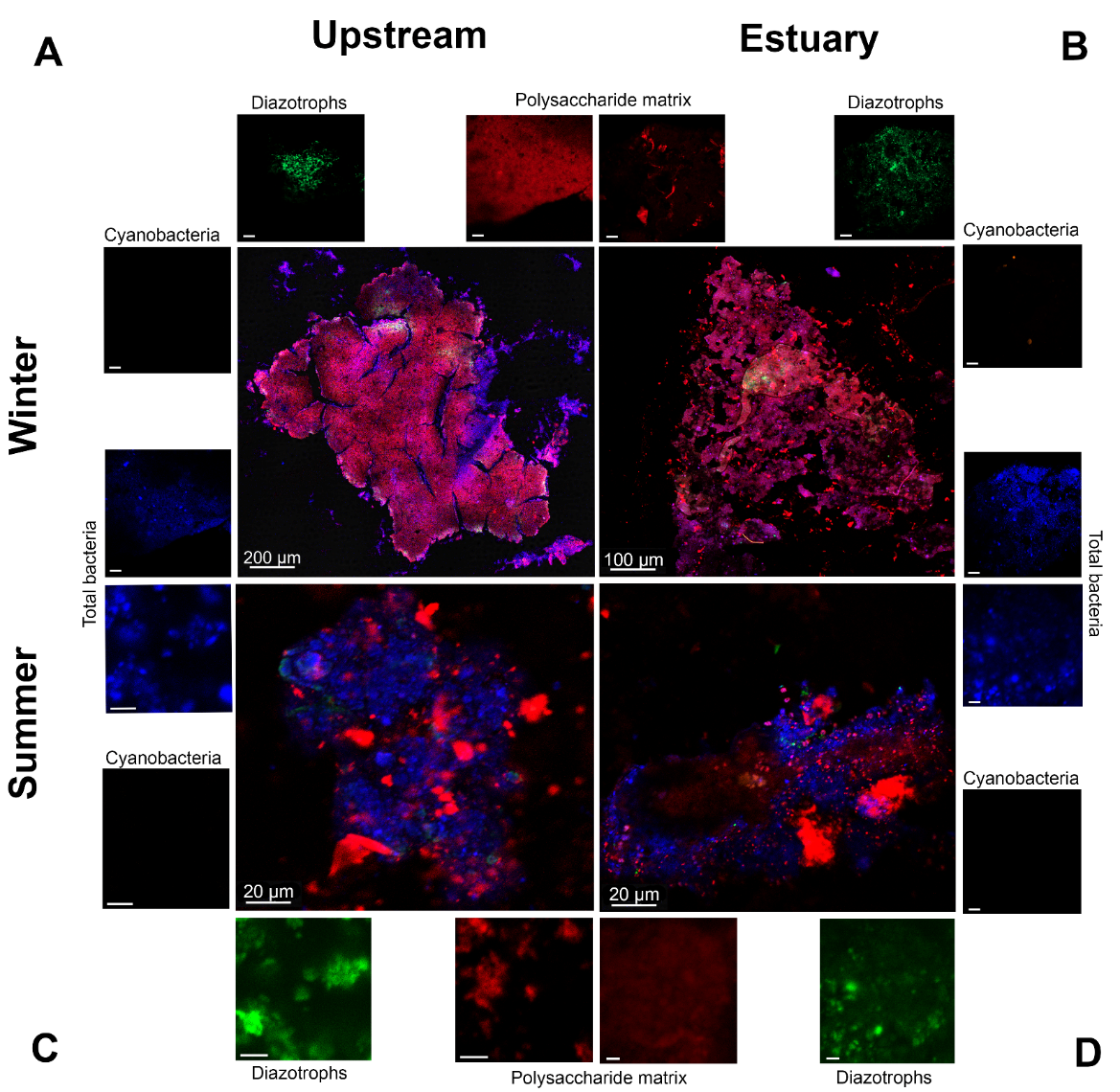


**Figure S1.** Immunolabeling micrograph of diazotrophs associated with aggregates collected from the Qishon River (stream and estuary). Images were captured by a confocal laser scanning microscope with a double close-up of bacteria associated with aggregates. Images were taken in four layers as cyanobacteria by autofluorescence of the phycoerythrin pigment (orange/white), diazotrophs by immunolabeling of nitrogenase (green), bacteria by DAPI (blue), and polysaccharides by ConA (red) The mark on the aggregates represents a close-up of the image. The reported scale bars in the top inserts are 10 μm (A) and 20 μm (B), while the scale bar in the bottom inserts is 2 μm.

**References**

Benavides, M., Martias, C., Elifantz, H., Berman-Frank, I., Dupouy, C., and Bonnet, S. (2018). Dissolved Organic Matter Influences N2 Fixation in the New Caledonian Lagoon (Western Tropical South Pacific). *Front. Mar. Sci.* 5, 1–11. doi:10.3389/fmars.2018.00089.

Bonnet, S., Dekaezemacker, J., Turk-Kubo, K. A., Moutin, T., Hamersley, R. M., Grosso, O., et al. (2013). Aphotic N2 Fixation in the Eastern Tropical South Pacific Ocean. *PLoS One* 8, e81265. Available at: doi:10.1371/journal.pone.0081265.

Hallstrøm, S., Benavides, M., Salamon, E., Evans, C., Potts, L., Granger, J., et al. (2021). Pelagic N2 fixation dominated by sediment diazotrophic communities in a shallow temperate estuary. *Environ. Microbiol.*, 1–15. doi:10.1002/lno.11997.

Heller, P., Tripp, H. J., Turk-Kubo, K., and Zehr, J. P. (2014). ARBitrator: a software pipeline for on-demand retrieval of auto-curated nifH sequences from GenBank. *Bioinformatics* 30, 2883–2890. doi:10.1093/bioinformatics/btu417.

Jabir, T., Vipindas, P. V., Jesmi, Y., Valliyodan, S., Parambath, P. M., Singh, A., et al. (2020). Nutrient stoichiometry (N:P) controls nitrogen fixation and distribution of diazotrophs in a tropical eutrophic estuary. *Mar. Pollut. Bull.* 151, 110799. doi:10.1016/j.marpolbul.2019.110799.

Li, D., Jing, H., Zhang, R., Yang, W., Chen, M., Wang, B., et al. (2020). Heterotrophic diazotrophs in a eutrophic temperate bay (Jiaozhou Bay) broadens the domain of N2 fixation in China’s coastal waters. *Estuar. Coast. Shelf Sci.* 242, 106778. doi:10.1016/j.ecss.2020.106778.

Pedersen, J. N., Bombar, D., Paerl, R. W., Riemann, L., and Seymour, J. R. (2018). Diazotrophs and N2 -Fixation Associated With Particles in Coastal Estuarine Waters. *Front. Microbiol.* 9, 1–11. doi:10.3389/fmicb.2018.02759.

Rahav, E., Bar-Zeev, E., Ohayon, S., Elifantz, H., Belkin, N., Herut, B., et al. (2013). Dinitrogen fixation in aphotic oxygenated marine environments. *Frointiers Microbiol.* 4, 1–11. doi:10.3389/fmicb.2013.00227.

Rahav, E., Giannetto, M. ., and Bar-Zeev, E. (2016). Contribution of mono and polysaccharides to heterotrophic N2 fixation at the eastern Mediterranean coastline. *Sceintific Reports* 6. doi:DOI: 10.1038/srep27858.
